# Surface Studies on Glass Powders Used in Commercial Glass-Ionomer Dental Cements

**DOI:** 10.3390/molecules26175279

**Published:** 2021-08-31

**Authors:** Agata Wawrzyńczak, Jacek Kłos, Izabela Nowak, Beata Czarnecka

**Affiliations:** 1 Faculty of Chemistry, Adam Mickiewicz University in Poznań, Uniwersytetu Poznańskiego 8, 61-614 Poznań, Poland; agata.wawrzynczak@amu.edu.pl (A.W.); jacek.klos@amu.edu.pl (J.K.); nowakiza@amu.edu.pl (I.N.); 2 Department of Biomaterials and Experimental Dentistry, Poznan University of Medical Sciences, Bukowska 70, 60-812 Poznań, Poland

**Keywords:** ionomer glasses, surface properties, density, particles morphology, thermogravimetric analysis

## Abstract

The surface properties of three commercial ionomer glass powders, i.e., Fuji IX, Kavitan Plus and Chemadent G-J-W were studied. Samples were analyzed by X-ray fluorescence spectroscopy (XRF), and the density was determined by gas pycnometry. Morphology was studied using scanning electron microscopy (SEM) and laser diffraction (LD) technique, whereas low-temperature nitrogen sorption measurements determined textural parameters like specific surface area and pore volume. Thermal transformations in the materials studied were evaluated by thermogravimetric analysis (TGA), which was carried out in an inert atmosphere between 30 °C and 900 °C. XRF showed that Fuji IX and Kavitan Plus powders were strontium-based, whereas Chemadent G-J-W powder was calcium-based. Powders all had a wide range of particle sizes under SEM and LD measurements. Specific surface areas and pore volumes were in the range 1.42–2.73 m^2^/g and 0.0029 to 0.0083 cm^3^/g, respectively, whereas densities were in the range 2.6428–2.8362 g/cm^3^. Thermogravimetric analysis showed that the glass powders lost mass in a series of steps, with Fuji IX powder showing the highest number, some of which are attributed to the dehydration and decomposition of the polyacrylic acid present in this powder. Mass losses were more straightforward for the other two glasses. All three powders showed distinct losses at around 780 °C and 835 °C, suggesting that similar dehydration steps occur in all these glasses. Other steps, which differed between glass powders, are attributed to variations in states of water-binding on their surfaces.

## 1. Introduction

Glass ionomer cements are widely used in dentistry with a variety of uses, including full restorations, liners and bases, luting agents, fissure sealants and adhesives for orthodontic brackets [1]. They have several attractive properties, including biocompatibility towards tooth tissue [2], fluoride release [3,4], inherent adhesion to the teeth [5,6] and low coefficient of linear thermal expansion [7].

Glass-ionomer cements are prepared from special ion-leachable glass powders. These are reacted with a solution of polymeric water-soluble acid [1,8,9,10], typically polyacrylic acid but also possibly acrylic/maleic or acrylic/itaconic copolymer or the copolymer of 2-methylene butanedioic acid with propenoic acid [10]. Commercial cements typically contain some of the polymeric acid mixed as a dry powder with the glass. This acid powder makes the effective concentration of the acidic polymer in the final mixed cement high without causing the cement liquid to be too viscous. High amounts of polymeric acid in the cement make the resulting cement strong [9], which is necessary to make it durable in clinical use.

The glasses used in glass-ionomer cements are made from complex mixtures, typically of calcium or strontium oxide, alumina, silica, aluminium phosphate and calcium or strontium fluoride [8,11]. These glasses are basic and react with the polymer to form a mixture of calcium (or strontium) and aluminium carboxylate salts as the cement sets [9]. There is also a secondary setting process involving inorganic species for the ion-depleted glass [12], the most important of which appears to be the phosphate [13]. The gradual formation of an inorganic network from these inorganic species is responsible for the changes that occur as the glass-ionomer matures, resulting in decreased plasticity, greater compressive strength and improved translucency [14].

Physicochemical properties of the surface of solids are a crucial aspect of the potential applications of various types of materials. In recent years, research has been conducted, among others, on icephobic strategies and obtaining materials with superwettability [15,16,17] and with a stable, damage resistant and omniphobic surface [18], proving that appropriately designed procedure allows designing materials with a strictly defined purpose.

**Scheme 1:**
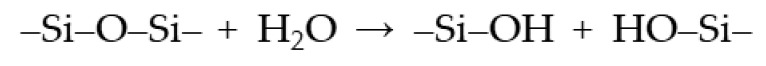
Hydrolysis of siloxane groups.

Recently, we have also investigated several aspects of the surface of glasses used in ionomer cements. Our studies have concentrated on the glass known as G338, which has the pre-firing composition listed in [19]. It is close in composition to glasses used in commercial ionomer cements, though their detailed composition may vary slightly, including the total replacement of the calcium salts with strontium ones. These studies [20] have shown that siloxane groups in the surface of the glass powder undergo hydrolysis as part of the setting reaction, generating silanol groups in the process shown in Scheme 1:

We have also shown that the glass G338 loses mass on heating but can regain only a fraction of this loss even under high humidity conditions [21]. The heat-treated glass sets quicker when mixed with polyacrylic acid than the as-received glass but gives a weaker set cement [21]. These findings suggest that heat treatment leads to the loss of surface silanol groups on the glass surface and the formation of siloxane groups.

Studying heat-treated G338 showed no morphological differences between as-received and heat-treated specimens using scanning electron microscopy. Furthermore, there were no significant differences between the measured values of specific surface area or pore volume between as-received and heat-treated glass powders. Thermogravimetric analysis up to 900 °C showed that mass loss occurred in four steps, two major and two minor. These losses are consistent with a four-step removal of water, the steps being (i) loss of loosely bound water from the surface, (ii) and (iii) minor but distinct step-wise loss of water hydrogen-bonded to the two distinct types of surface silanol groups, and (iv) dehydration of silanol groups to form -Si-O-Si- linkages in the surface as previously mentioned [19].

In the present paper, we extend our studies to glass powders used in commercial glass-ionomer cements. Three materials are studied. Samples were examined before heat treatment using SEM to evaluate the shape and size of glass particles. Additionally, particle size measurements were based on laser diffraction. Specific surface area and pore volume were also determined. This has allowed us to extend our knowledge of the surface behavior to commercial ionomer glass powders and gain further insight into the surface structure and composition of these powders.

Our research aims to study the basic physical and chemical properties of components of glass-ionomer cements, namely, glass powders. These properties, which we present for the first time, are not provided by manufacturers, and we try to fill this gap in information. This basic information could be valuable to design further research on glass-ionomer cements properties, such as compressive strength, hardness, solubility or ion release studies. Our findings can help adequately interpret and understand the relationship between materials’ basic properties and results obtained in advanced laboratory tests performed by other researchers.

To the best of our knowledge, such comparative studies have not yet been conducted, and the knowledge on the surface physicochemistry of the glass powders will undoubtedly contribute to a broader understanding of their performance in commercial glass-ionomer dental cements.

## 2. Results and Discussion

**Figure 1:**
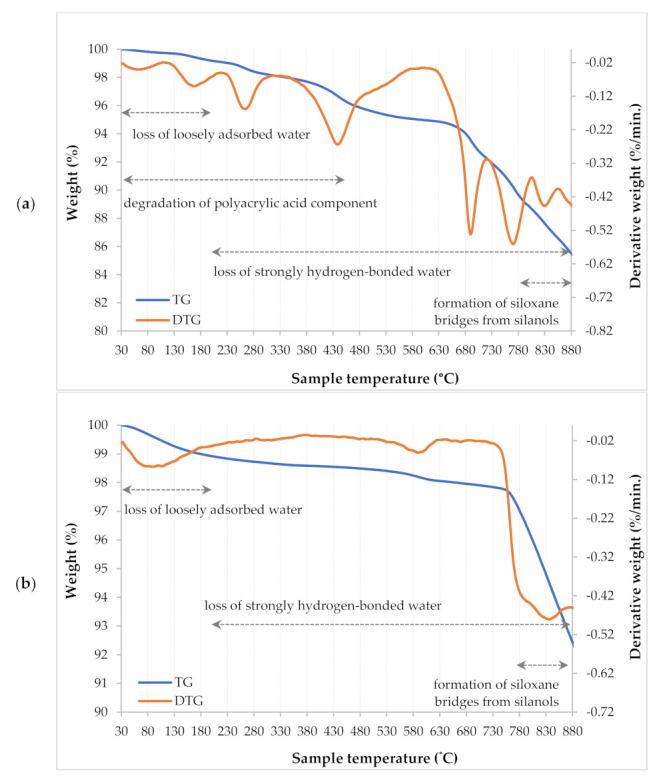

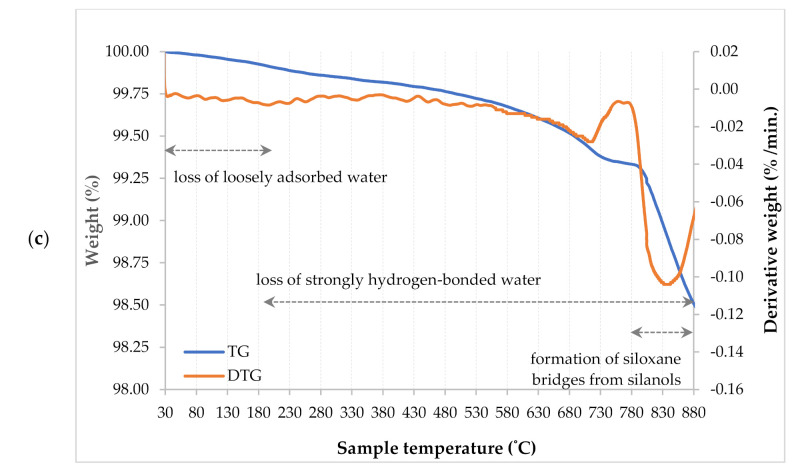
Thermogravimetric trace (30–880 °C) for (**a**) Fuji IX glass; (**b**) Kavitan Plus glass; (**c**) Chemadent G-J-W glass (TG—thermogravimetry, DTA—derivative thermogravimetry).

Thermogravimetric results for each glass are shown in Figure 1, and the list of distinct peaks in the differential plots for each glass powder are given in Table 1.

The thermogravimetric results show that there is a considerable mass loss on heating of all three powders and that this mass loss generally occurs in a series of discrete steps. Fuji IX glass powder showed the highest number of steps, starting from 60 °C and going upwards. This particular glass powder is supplied pre-mixed with some dried polyacrylic acid powder, and some of the observed peaks can be attributed to the decomposition of the polymer component. Thermal decomposition of polyacrylic acid is known to yield a variety of products, with anhydride formation occurring first [22] and later proceeding to the evolution of CO_2_ and a range of volatile organic fragments [23]. A previous thermogravimetric study of polyacrylic acid showed a clear peak at 260 °C [24], which corresponds closely to our observed peak at 260 °C. This study also reported peaks at 200 °C and 390 °C, which were not observed in our results. However, there were peaks at 170 °C and 440 °C, which might have similar origins and might be shifted relative to those previously observed by differences in the interactions with the solid components. The previous study involved an alumina-filled polyacrylic acid composite, and the intimate mixing in that material might have altered the energetics of specific decomposition processes. Whatever the explanation, it is difficult to attribute all of the peaks in the thermogram of Fuji IX glass powder, except to assign them generally to degradation steps of the polyacrylic acid component.

**Scheme 2:**
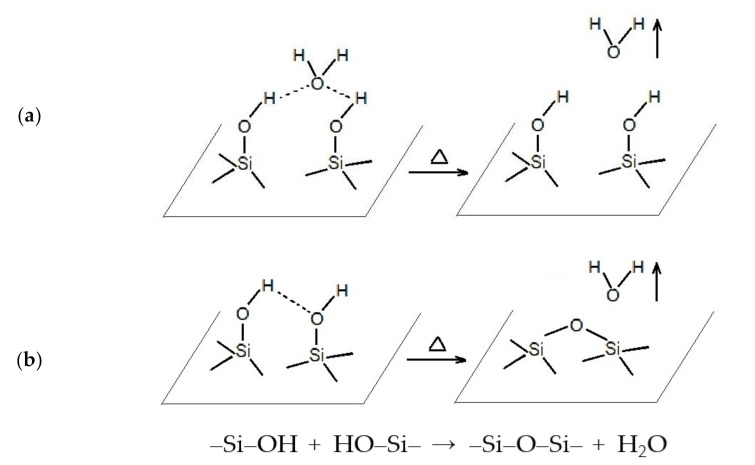
Removal of water in the glass surface: (**a**) water bounded to the surface; (**b**) condensation of pairs of silanol groups.

Other peaks arise from losses from the inorganic glass component, as they do in both Kavitan Plus and Chemadent G-J-W glass powders. Peak patterns are generally different between the glasses, except for the final two, at 775–780 °C and around 835 °C, which occur in all three glasses. They were also observed previously for G338 [19]. The losses are attributed to the removal of water, either pre-existing water in various states of interaction with the surface of the glasses particles or water arising from the condensation of pairs of silanol groups in the glass surface. These processes may be described according to Scheme 2:

This reaction was previously assumed to occur in the experimental glass G338 at the highest temperature at which a peak was observed in the differential thermogram, i.e., 834 °C [19]. Previous studies of various silica-based materials have shown that there are several distinct ways in which water can be lost on heating, starting with simple loss of adsorbed water layers and going on to involve the creation of siloxane groups by dehydration of silanols [25,26,27]. In thermogravimetry, it has been suggested that peaks from room temperature up to 200 °C are due to loss of adsorbed water from silica surfaces [26]. These surfaces are known to have water adsorbed onto them in several different ways, ranging from loosely bound films to relatively strongly bound water molecules, attached to specific silanol groups at the surface by hydrogen bonds [25,27].

There is some dispute about how many distinct types of silanol groups there may be at the surface. Sulpizi et al. [28] identified two, namely, in-plane and out-of-plane for the silica surface. By contrast, Cerveny et al. [26] claimed three, namely, isolated, vicinal and germinal. Whatever the number, we may presume that each type can be present to a greater or lesser extent, depending on the composition of the silica-based substrate, and that these silanols may vary (a) in the extent to which they can hydrogen-bond with individual water molecules and (b) in their ability to react with each other to generate siloxane bridges at the surface by dehydration.

In our previous work, we noted the occurrence of four peaks in the differential thermogram of G338 and attributed these to a four-step loss of water from the glass powder surface [19]. These steps involved initial loss of a loosely bound water film, the loss of hydrogen-bonded water from two different types of surface silanol, and lastly, dehydration of adjacent silanol groups to form siloxane bridges. We currently see no need to revise this suggestion, though we note that the commercial glasses in the current study show different patterns of water loss. Like G338, Kavitan Plus glass shows four reasonably distinct peaks, but the lower two of them occur at different temperatures from those shown by G338. Chemadent G-J-W glass shows a long gradual loss of mass up to about 700 °C, suggesting loss of loosely bound water from a very wide range of possible sites on the surface.

In all cases, both in this study and in our previous one on G338 [19], there are two peaks at very high temperatures, namely, around 780 °C and around 835 °C. We previously attributed the latter to the formation of siloxane bridges in the surface, an assignment made because the high temperature at which the loss occurs indicated that a highly endothermic process was involved. The formation of siloxane from silanols seems likely to be just such an endothermic process. We attributed the peak at around 780 °C to loss of strongly hydrogen-bonded water [19]. However, it is possible that, since this is also a highly endothermic process, it might also be due to a silanol condensation process, perhaps involving silanol groups that are better oriented to undergo the process and which require slightly less energy to do so.

Whatever the details of the mass loss reactions from ionomer glasses, we attribute them to various water loss processes. Lower temperature losses seem to arise from the loss of loosely bound water that occurs on the surface as a film of pre-existing water. Higher temperature losses seem to occur when distinct groups of hydrogen-bonded water molecules are lost from the surface. Finally, at the highest temperature(s), there is at least one loss due to the formation of siloxane bridges from pairs of silanol groups. Differences we attribute to the fact that the ionomer glasses have different compositions, which in turn cause there to be different numbers and distributions of silanol groups on the particle surfaces. Finally, we note that two high-temperature losses, at around 780 °C and around 835 °C, appear characteristic of ionomer glasses.

**Figure 2:**
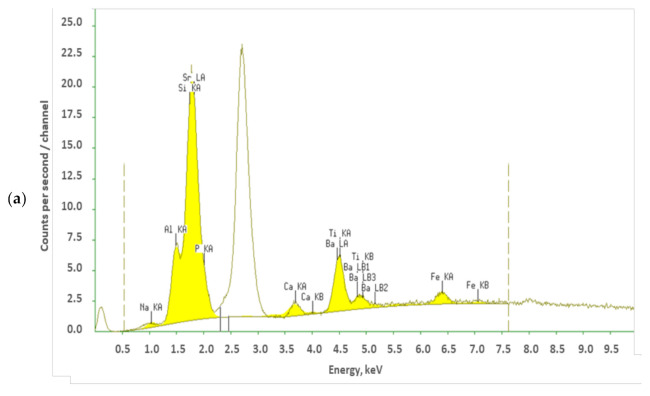

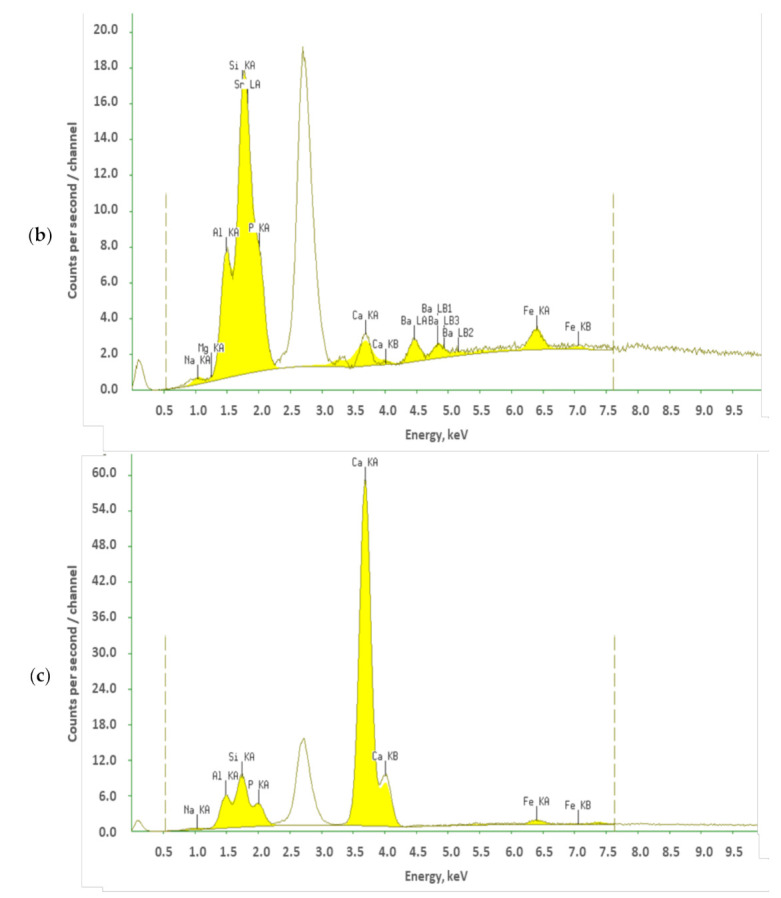
XRF spectrum (13 kV) of (**a**) Fuji IX glass; (**b**) Kavitan Plus glass; (**c**) Chemadent G-J-W glass.

Figure 2 and Table 1 show the X-ray fluorescence spectra of the glasses. They prove that Fuji IX and Kavitan glasses were strontium-based, whereas Chemadent glass was calcium-based. Manufacturers of dental materials are not required to provide information on the detailed composition of their products, so investigation of this composition is of great importance for researchers who study their other physicochemical properties.

XRF results showed that two of the glass powders, namely, Fuji IX and Kavitan Plus, were strontium-based (Figure 2a,b). By contrast, Chemadent G-J-W glass powder was found to be calcium-based (Figure 2c). The substitution of calcium with strontium in these glasses is well established and makes little difference to the setting chemistry. Both calcium and strontium can form divalent ions and crosslink polyacrylic acid molecules [1,9,29]. Strontium has the advantage of making the set cement opaque to X-rays so that a dental filling made from the cement is clearly visible when teeth are examined radiographically in the clinic.

Fuji IX and Kavitan Plus glass powders both contain traces of calcium, and Fuji IX contains a small amount of barium, presumably present as a radio-opacifier. Fuji IX contains a small amount of titanium, probably present as part of the pigment system. All three glasses have traces of iron, which is also probably part of the pigment system and is present as iron oxide. All glasses also contain reasonable amounts of silicon, aluminium and phosphorus, all of which are typically used to fabricate ionomer glasses and are known to have distinctive structural roles [8,9].

**Figure 3:**
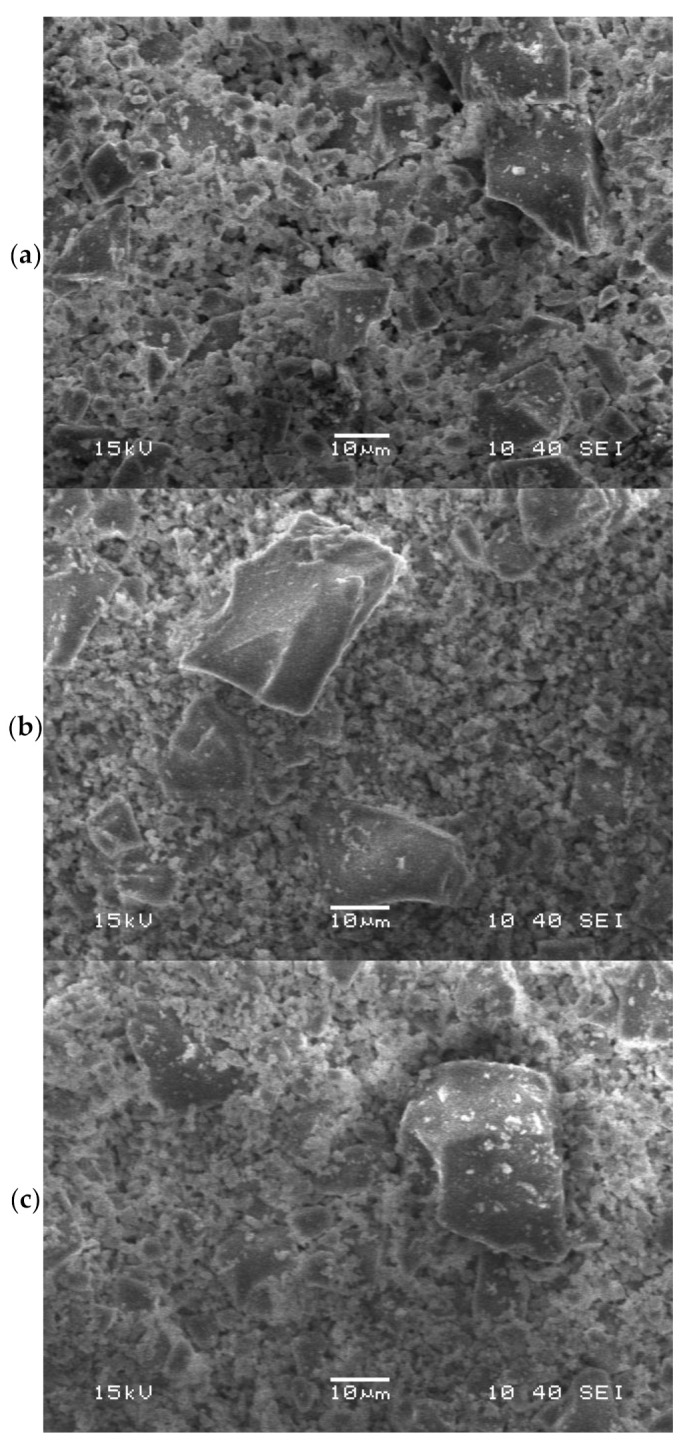
SEM image of (**a**) Fuji IX glass; (**b**) Kavitan Plus glass; (**c**) Chemadent G-J-W glass.

Figure 3 shows SEM images of the powders. There is a range of particle sizes in all cases, with some large particles but with a majority of particles being much smaller. Particles were angular in shape rather than smooth and rounded.

**Figure 4:**
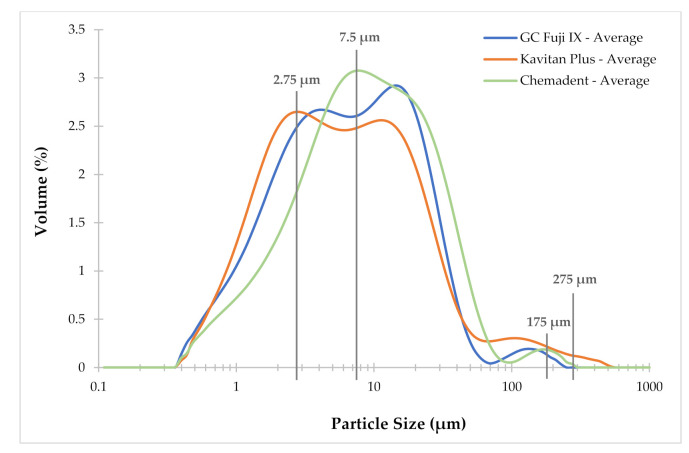
Particle size distributions in the three ionomer glass powders (average of five measurements).

The SEM images of the powders show them all to have similar appearances. These glasses typically have a broad range of particle sizes, with most particles being small but with occasional large irregular particles in the mixture. These results fully agree with those of the laser diffraction technique, where the PSDs (Figure 4) show a major contribution from particles with sizes between 0.4 μm and 70 μm, with a small contribution from larger particles.

Data on particle sizes of ionomer glasses are also shown in Table 2 and Figure 4. Values were comparable for all three glass powders, but those for Chemadent G-J-W were generally slightly larger than for the other two powders.

Particle sizes were similar for all three powders, though Chemadent G-J-W showed slightly larger values, indicating that even the smallest fraction of the powder had larger particles than the other two glasses (Table 2). In the laser diffraction measurements, the definition of the particle size is based on the concept of equivalent spheres. In other words, the reported particle size is that of an equivalent sphere having the same volume or mass as the actual particle. Because of this, different parameters can be used for the characterization of the particle population within the sample, depending on the information we want to obtain.

The D[3,2] parameter is the most sensitive to the presence of fine particulates, and this shows Chemadent G-J-W to have the highest average diameter (4.07 μm) in the fraction of smaller particles. The average diameter D[4,3] considers mainly particles with larger diameters, and this parameter was the highest for Kavitan Plus (15.44 μm). Particle size distribution plots are shown in Figure 4, where the curve for Kavitan Plus reaches values higher than 275 μm, confirm this. Nevertheless, the median d(0.5) of the particle size distribution shows that the average particle size for Kavitan Plus is the smallest due to a significant amount of particles of the finest size. The first maximum on particle size distribution curve occurs for particles of sizes close to 2.75 μm, which is the smallest amongst all curves. On the other hand, the particle size distribution curve for Chemadent G-J-W shows the lowest contribution of particles with the smallest sizes and two additional maxima at values of 7.5 μm and 175 μm. Overall, this gives 8.22 μm as the median value for this powder, the highest of all the ionomer glasses studied. The results obtained with the laser diffraction technique were in good agreement with those from SEM, where images showed the mixtures consisted of mainly small particles with occasional large particles also present.

The densities of the glass powders are shown in Table 3. Glasses were found to differ to significant extents, ranging from 2.6428 g/cm^3^ to 2.8362 g/cm^3^.

Powder densities were found to vary somewhat, lying between 2.6428 g/cm^3^ to 2.8362 g/cm^3^. These results are comparable with that obtained for the experimental glass G338 (2.6438 g/cm^3^) [19]. Apart from our earlier report, there seem to have been no other results published on the densities of these glasses.

Results for low-temperature nitrogen sorption are also presented in Table 3. These showed that both the specific surface areas and the pore volumes varied widely. The Kavitan Plus glass powder had the largest specific surface area (2.73 m^2^/g) and also the largest pore volume (0.0083 cm^3^/g), both of which differed from those of the other glass powders to extents that were statistically significant (*p* > 0.001). By contrast, the differences between these features for the Fuji IX and Chemadent G-J-W glasses were not significant. Specific surface area values for this type of glass powder have been reported only infrequently. As expected, the largest specific surface area (2.73 m^2^/g), found for the glass Kavitan Plus, also had the largest pore volume (0.0083 cm^3^/g). Our results are comparable to those previously reported for this type of glass. Todo et al. [30] for example, reported a series of glass powders with specific surfaces areas in the range 2.50–4.00 m^2^/g, and Crowley et al. [31] reported glass in the range 1.76–4.36 m^2^/g, with most being close to 2.00 m^2^/g, a result confirmed by our data. A recent report on an experimental strontium-based glass gave a value of 0.73 m^2^/g [32], which our results show to be low for a practical ionomer glass.

## 3. Materials and Methods

Studies were carried out on the three commercial ionomer glass powders, namely, Fuji IX (GC, Tokyo, Japan), Kavitan Plus (Kerr, Brea, CA, USA) and Chemadent G-J-W (Chema, Rzeszów, Poland). The selection of the cements was based on the type of glass-ionomer cement. All tested materials are examples of the same group of permanent restorative dental glass-ionomer cements; however, they are produced by different manufacturers from different countries and continents. Moreover, according to manufacturers, the powders of these selected cements are available in the form of pure glass powder. Other types of glass-ionomer cement powders are usually a mixture of ionomer glass with vast amounts of poly(acrylic acid) or other polymer acids. In all cases, the compositions were determined by X-ray fluorescence spectroscopy at 13 kV using a MiniPal spectrometer (Malvern Panalytical BV, Almelo, the Netherlands). Determinations were carried out in an atmosphere of helium gas, and the radiation source was an X-ray tube with a rhodium cathode.

The density of all three glass powders was measured by gas pycnometry using an Ultrapyc 1200e pycnometer (Quantachrome Instruments, Boynton Beach, FL, USA) and helium gas. Thermogravimetric analyses were carried out in the temperature range 30–900 °C using a Pyris TGA1/GC/MS Clarus 680 SQ8 machine (Perkin Elmer, Waltham, MA, USA). The chosen temperature range was based on the protocol employed for SiO_2_ and Al_2_O_3_ testing by the analogy to investigations of SiO_2_ and Al_2_O_3_, especially the higher limit of 900 °C, at which the surface of Al_2_O_3_ is deprived of hydroxyl groups [19]. The temperature was ramped at 10 °C/min, and the samples were kept under an atmosphere of helium gas flowing at 40 mL/min. Scanning electron micrographs were recorded for all three original glass powders using a JEOL JM-6380LA instrument (JEOL Ltd., Tokyo, Japan).

Glass particle sizes were measured with laser diffraction (LD), using a Mastersizer 2000 (Malvern Instruments Co., Ltd., Malvern, UK) device with distilled water as the dispersion medium. Measurements were repeated five times and averaged for each sample. Based on the laser diffraction measurements, particle size distributions (PSD), where the volume of particles is displayed as a function of the particle size, were plotted and the following parameters calculated:

- D[3,2] (μm)—Σd^3^/Σd^2^; the surface area mean (Sauter Mean Diameter); this value is the most sensitive to the presence of fine particulates in PSD;
- D[4,3] (μm)—Σd^4^/Σd^3^; the volume moment mean (De Brouckere Mean Diameter); this value is the most sensitive to the presence of large particulates in PSD;
- d(0.1) (μm)—10% of the particles have diameters smaller than this value;
- d(0.5) (μm)—50% of the particles have higher diameters and 50% lower diameters than this value (median of PSD);
- d(0.9) (μm)—90% of the particles have diameters smaller than this value.

Finally, low-temperature nitrogen sorption was used to determine the specific surface area (BET) and pore volume on the glass powders degassed at 28 °C. Eight individual powder samples were used per material, and means and standard deviations were determined. Differences were tested for significance using the Student *t*-test. Measurements were performed with NOVA 1200e sorptometer (Quantachrome Instruments, Boynton Beach, FL, USA).

## 4. Conclusions

This study of the properties of three commercial ionomer glass powders has shown that they have a wide range of particle sizes and densities in the range 2.6–2.9 g/cm^3^. Specific surface areas lay between 1.42 and 2.73 m^2^/g and pore volumes between 0.0029 and 0.0083 cm^3^/g. In both cases, Chemadent G-J-W glass gave the lowest values and Kavitan Plus the highest. All tested samples are characterized with quite a broad particle size distributions, with Kavitan Plus having the broadest range of particle sizes and the highest contribution of smaller particles.

The XRF results showed that Fuji IX and Kavitan Plus glass powders were strontium-based, whereas Chemadent G-J-W glass powder was calcium-based, and thermogravimetric analysis showed that the glass powders underwent a series of steps leading to loss of mass. Fuji IX powder had the highest number of loss steps, and some of these are attributed to the dehydration and decomposition of the polyacrylic acid present in this powder. The other glasses showed different mass losses, with fewer steps. These are broadly attributed to three processes: loss of loosely bound water films, loss of strongly bound hydrogen-bonded water, and dehydration of surface silanol groups leading to the formation of siloxane bridges in the surface. The exact orientation and density of these surface silanol groups appear to vary with glass composition, which is why the loss patterns differ. However, all glasses showed distinct losses at around 780 °C and 835 °C, suggesting that all glasses studied undergo two similar dehydration steps, and these seem to be characteristic of ionomer glasses.

## Figures and Tables

**Table 1:** Peak positions in differential thermogravimetric traces and elemental composition of glass powders from XRF.

Glass Powder	Elements (XRF)	Peak Positions/°C (TGA)
Fuji IX GP	Na (trace), Sr, Si, Al, P, Ca (trace), Ba, Ti (trace), Fe (trace)	60, 170, 260, 440, 700, 775, 830
Kavitan Plus	Na (trace), Sr, Si, Al, P, Ca (small amount), Fe (trace)	80, 580, 780, 825
Chemadent G-J-W	Na, Si, Al, P, Ca, Fe (trace)	30–700 (broad loss), 750 (very slight), 805, 830

**Table 2:** Particle size data from laser diffraction measurements (standard deviations in parentheses).

Glass Powder	D[3,2] ^1^ (μm)	D[4,3] ^2^ (μm)	d(0.1) ^3^ (μm)	d(0.5) ^4^ (μm)	d(0.9) ^5^ (μm)
GC Fuji IX	3.20 (0.03)	11.32 (0.35)	1.29 (0.02)	6.17 (0.04)	24.03 (0.30)
Kavitan Plus	3.01 (0.01)	15.44 (0.36)	1.21 (0.00)	5.46 (0.01)	26.85 (0.42)
Chemadent G-J-W	4.07 (0.01)	13.62 (1.14)	1.68 (0.01)	8.22 (0.05)	30.61 (0.98)

^1^ D[3,2] (μm) = Σd^3^/Σd^2^; ^2^ D[4,3] (μm) = Σd^4^/Σd^3^; ^3^ d(0.1) (μm)—10% of the particles have diameters smaller than this value; ^4^ d(0.5) (μm)—50% of the particles have higher and 50% lower diameters than this value; ^5^ d(0.9) (μm)—90% of the particles have diameters smaller than this value.

**Table 3:** Details of commercial ionomer glass powders (standard deviations in parentheses).

Material	Manufacturer	Density (g/cm^3^)	Specific Surface Area (m^2^/g)	Pore Volume (cm^3^/g)
Fuji IX GP	GC, Japan	2.8362 (0.0077)	1.53 (0.23)	0.0039 (0.0010)
Kavitan Plus	Kerr, USA	2.7763 (0.0048)	2.73 (0.66)	0.0083 (0.0014)
Chemadent G-J-W	Chema, Poland	2.6428 (0.0148)	1.42 (0.32)	0.0029 (0.0010)

## Data Availability

Not available.

## Sample Availability

Samples of the Fuji IX glass, Kavitan Plus glass, and Chemadent G-J-W glass are available from the authors.
